# The Risk of CVDs from Desalinated Seawater: A Nested Case-Control Study

**DOI:** 10.3390/ijerph19127422

**Published:** 2022-06-16

**Authors:** Juexin Shi, Yuan Liu, Qin Wang, Xiaojian Hu, Bixiong Ye, Shaoxia Dong

**Affiliations:** National Institute of Environmental Health, Chinese Center for Disease Control and Prevention, Beijing 100021, China; shijuexin1998@163.com (J.S.); yuan_liu90@163.com (Y.L.); wangqin@nieh.chinacdc.cn (Q.W.); huxiaojian@nieh.chinacdc.cn (X.H.); yebixiong@nieh.chinacdc.cn (B.Y.)

**Keywords:** desalination, cardiovascular disease, incidence

## Abstract

The aim of this paper was to assess the association between desalinated seawater and cardiovascular diseases (CVDs). We conducted a nested case-control prospective study on a cohort of 7806 subjects who live on an island of China that lacks fresh water. From this cohort, we identified 140 paired CVD cases and matched controls by sex and age during the same period. Questionnaires were used in order to investigate basic sociodemographic information and risk factors for CVDs, and urine samples were collected to measure calcium and magnesium levels. Using these data we developed and tested both univariate and multivariate logistic regression models. We observed no significant differences in risk of CVDs between groups with desalinated seawater and fresh water intake. From multivariate logistic regression, we found that obesity (OR = 5.38, 95% CI: 1.05–27.45), physical activity (OR = 0.35, 95% CI: 0.16–0.75), hypertension (OR = 3.61, 95% CI: 1.58–8.25), alcohol consumption (OR = 2.57, 95% CI: 1.02–6.47), and irritability (OR = 4.30, 95% CI: 1.93–9.60) were associated with an increased risk of CVD. In this population, we found no association between desalinated seawater intake and CVDs; the incidence of CVDs was primarily related to lifestyle.

## 1. Introduction

With both increases in global population and environmental pollution, safe water sources around the world are becoming less available. According to the United Nations World Water development report 2018: Nature-Based Solutions for Water (Executive Summary), about 3.6 billion people (more than half the global population) live in areas that potentially lack water resources for at least one month per year, and this population could increase to some 4.8–5.7 billion by 2050 [1]. As one of the most water-deficient countries in the world, China’s average water resources per capita is only one-quarter of that of the rest of the world [2]. As an expanding resource brought about by new technology, desalinated water can effectively alleviate this shortage of fresh water. So far, the technology has been vigorously promoted and applied in many countries around the world.

In the Middle East and North Africa, for example, desalinated water has already been incorporated into municipal water supplies as the major source of water. In addition, the installed capacity of seawater desalination in these areas accounts for 48% of the world’s total desalination [3]. Some cities [4,5,6] have also successively carried out seawater desalination projects to cope with their own water resource crises. Recently, though, China has aggressively developed its desalination industry. According to China’s 2019 *National Seawater Utilization Report*, there were 115 desalination projects producing 1.5 million tons of fresh water daily by the end of 2019 [7].

Seawater desalination involves three phases: pretreatment, desalination, and post-treatment. This 3-phase process removes most of the toxic substances in seawater such as microorganisms and organic matter and also reduces salt volume. Currently, mainstream seawater desalination technologies include multistage flash distillation (MSF), reverse osmosis (RO), and low-temperature multi-effect distillation (MED). However, the desalination process not only removes harmful substances from seawater but also removes some beneficial substances, such as calcium, magnesium, fluorine, and other elements, resulting in a low hardness of desalinated water. Israeli studies have shown that desalinated seawater in Kibbutz Maagan Michael contains 0.8 mg/L Mg, and 34.0 mg/L Ca which significantly lower than Mg and Ca levels of Hadera (20.5 mg/L and 94.2 mg/L, respectively) and Beer Sheva (18.5 mg/L and 50.7 mg/L, respectively) in fresh water [8]. 

Cardiovascular diseases (CVDs) are the leading cause of death globally; an estimated 17.9 million people died from CVDs in 2019, representing 32% of all global deaths [9]. There are many risk factors for CVDs including hypertension, dyslipidemia, diabetes, smoking, excessive drinking, and obesity. In addition, many studies have shown that higher calcium and magnesium concentrations can reduce the incidence of CVDs. Momeni et al. revealed that a higher calcium content was associated with a reduced number of CVDs in Khansar County in Isfahan province, Iran [10], and Kousa et al. investigated the acute myocardial infarction incidence among patients aged 35–74 years in rural Finland and found that the incidence of acute myocardial infarction decreased by 2% for every 1 mg/L increase in water magnesium concentration [11]. In 2003, scientists in Israel found that drinking water with a Mg concentration of 10–40 mg/L or higher can reduce coronary heart disease (CHD) mortality by 30–35% [12] and in 2016, a meta-analysis indicated that higher magnesium levels were associated with lower CHD mortality risk. (RR = 0.89, 95% CI: 0.79–0.99) [13]. Additionally, some studies have shown that there is no relationship between the hardness of water and CVDs. Leurs et al. showed that there was no relationship between hardness and ischemic CVDs or stroke when exposure assessment was carried out by hardness classification [14], and the results of prospective studies by Morris et al. showed that higher hardness could not significantly prevent CHDs or CVDs and neither could higher calcium nor higher magnesium [15].

Compared with common water resources, desalinated water has higher purity and lower hardness; the total hardness of desalinated water by reverse osmosis technology is lower than 75 mg/L, the magnesium concentration is less than 7 mg/L, and the calcium concentration is less than 6 mg/L [16,17], which are far lower than the limiting values described in China’s *Hygienic Standard of Mineralization for Drinking Water with Low Mineral Level[s]* (GBJ 1335-92) [18]. Despite such findings, there is no research that specifically clarifies the association between hardness, calcium, and magnesium and the incidence of CVDs. Hence, this study evaluated the association between desalinated water and CVDs using a nested case-control study, specifically discussing the effects of calcium and magnesium in drinking water on CVDs. Further, the study analyzes the broad risk factors of CVDs in coastal residents and lays a theoretical foundation for following studies about the interaction between different risk factors in desalinated seawater and CVDs. Finally, this study provides basic data for future health risk assessments of desalinated seawater. The novelty of this paper is to explore the risk between desalinated seawater and CVDs by the nested case-control study which is better than previous case-control studies and cohort studies in both efficiency and demonstration ability. At the same time, we analyzed the differences in Ca and Mg levels in urine in different groups from the two perspectives of exposure and outcome, respectively. As well, it provides a theoretical basis for subsequent study.

## 2. Population and Methods

### 2.1. Study Sites

**Figure 1:**
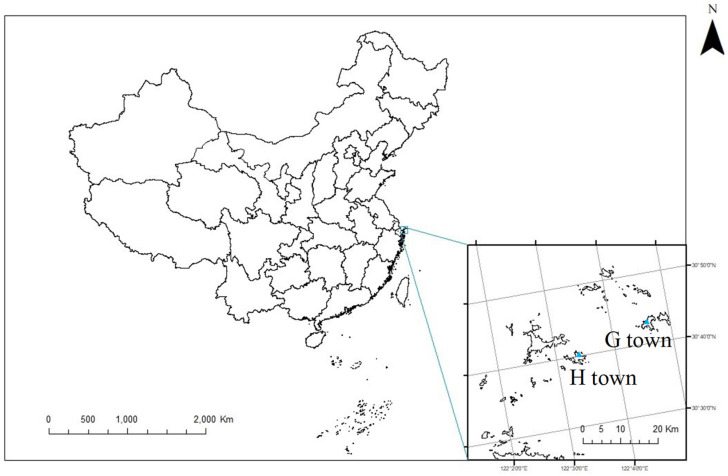
The study sites of Huang Long Town (H town) and Gou Qi Town (G town) in Shengsi County.

We selected Shengsi County as the study site, which is located in the northernmost part of Zhoushan City and the easternmost part of Zhejiang Province in China (Figure 1). Zhejiang Province is located in the east of Hangzhou Bay and the southeast of the Yangtze River estuary, between the northern latitudes 30°24′ and 31°04′ and eastern longitudes 121°30′ and 123°25′. Shengsi County consists of 404 islands, 16 of which are inhabited. It covers an area of 8824 square kilometers, including 86 square kilometers on land and 8738 square kilometers on the sea. Shengsi County was one of the earliest areas in China that developed desalination projects and has used desalinated seawater as one of the primary sources of drinking water since 1997.

Shengsi County has 3 towns and 4 townships, and this study takes two towns with a similar population base and different water resources as study sites. Huang Long Town (H town) had a registered population of 9046 in 2016. In addition, 8349 residents were registered in Gou Qi Town (G town), which is further away from the county center than H town. Due to its severe shortage of freshwater resources, G town built a desalination plant in 2009 that consistently provides desalinated water for residents. In contrast, H town has abundant fresh water that is stored in local reservoirs, and our previous unpublished studies have indicated that the water total hardness is significantly different between G town (<5 mg/L) and H town (140 mg/L). An early study has shown that the total hardness of fresh water and desalinated seawater was obviously different in Shengsi County [19].

### 2.2. Study Design and Population

We conducted a nested case-control study based on a previous cohort in 2013 that was approved by the Ethics Committee of the National Institute of Environment Health, China CDC (Protocol ID: 2017018). Residents living in the island areas for more than 10 years were selected for the baseline population, and they have undergone complete physical examinations and chronic disease surveillance during follow-up periods beginning in 2013. The participants who were previously diagnosed with CVDs and/or stroke were excluded, and we also excluded individuals with incomplete follow-up information. In total 7806 participants were enrolled in the study.

During the period from 1 January 2013 to 31 December 2016, 147 new cases diagnosed with CVDs and stroke were enrolled in the case group. A gender and age matched control without CVDs and stroke was selected randomly with a ratio of 1:1 in the baseline population, where the matching rule required the same gender and an age gap of no more than 2 years. Finally, 140 pairs of participants were investigated in this study with a loss to follow-up of 4.8%.

### 2.3. Field Investigation

The Field investigation included information surveyed by questionnaire and urine sample collection. Before the survey, subjects were informed of the study content in detail, and they agreed to participate in the investigation and signed informed consent forms.

#### 2.3.1. Questionnaire

The subjects filled out this study’s questionnaire with the help of professionally trained investigators. The questionnaire contains four parts: (1) General information, including gender, age, ethnicity, marital status, education, and occupation; (2) Health status, including height, weight, medical history of CVDs, stroke, and other diseases, and family medical history; (3) Drinking water and dietary habits, including the acceptance of desalinated seawater, water consumption, and household salt and oil use; and (4) Living habits and mental health, including smoking, exposure to second-hand smoke, alcohol use, tea drinking, physical activities, and psychological inquiries.

We defined BMI ≥ 24 as overweight and BMI ≥ 28 as obesity. Without taking antihypertensive drugs, systolic blood pressure ≥ 140 and/or diastolic blood pressure ≥ 90 mmHg were measured three times on different days, which could be diagnosed as hypertension. With taking antihypertensive drugs, the patient who has a history of hypertension was still diagnosed with hypertension, although blood pressure was <140/90 mmHg. Fast blood glucose ≥ 7.0 mmol/L or the patient who is undergoing diabetes treatment was defined as diabetes. We defined the following conditions as hyperlipidemia: total cholesterol ≥ 5.2 mmol/L (or 200 mg/dL); triglyceride ≥ 1.7 mmol/L (or 150 mg/dL); HDL-C < 1.0 mmol/L (or 40 mg/dL). Smoking was defined as continuous or cumulative smoking for 6 months or more in a lifetime. Secondhand smoke is defined as smoke exhaled by the smoker and exhaled from the end of the cigarette.

#### 2.3.2. Urine Sample Collection

After finishing the questionnaire, subjects received urine cups and zip-locked bags from investigators and were informed how to collect their own urine samples. The following day 30 mL of a midstream specimen of the morning’s urine was collected from each subject and stored at 4 °C. The samples were then sent back to the laboratory by a cold chain in order to measure urinary creatinine, calcium, and magnesium levels. All tests were provided by Beijing minsheng sino assessment group Co., Ltd., Beijing, China.

### 2.4. Quality Control

A pilot investigation was conducted. Qualified investigators were responsible for introducing and filling in questionnaires after professional training, and the coincidence rate of random inspection on the questionnaire was 95%. In order to ensure the accuracy of the results, we implemented double entry and logical validation methods. Urine analysis reports were issued by a third-party inspection agency with China Inspection Body and Laboratory Mandatory Approval (CMA) and China National Accreditation Service for Conformity Assessment (CNAS) qualifications. Quality control during urine creatinine test: (1) The creatinine standard reserve solution was prepared before the experiment. As well, standard series was configured with it by doubling dilution; (2) The chromogenic agent was mixed before each batch of the samples was used; (3) Each batch of samples: Correlation coefficient r > 0.998. Two blanks detection, and the blank absorption value A_0_ < 0.002. No less than 10% of parallel samples, and relative error < 10%; (4) A standard recovery experiment was conducted for each 15 to 20 samples, and at least one standard recovery experiment was conducted for each batch. The recovery rate was controlled between 90% and 110%.

### 2.5. Statistical Analysis

All statistical analysis was conducted using SAS, version 9.3. Continuous variables were tested using independent-samples *t*-tests, and Chi-squared tests were used to compare categorical data in different groups. The total number of cases ≥ 40, a theoretical frequency ≥ 1 and <5, *χ*2 test results to the Continuity Correction; the total number of cases ≥ 40, at least 2 theoretical frequencies ≥ 1 and <5, see Fisher’s Exact Test results; the total number of cases < 40 or theoretical frequency < 1, see Fisher’s Exact Test results. Univariate analysis and multivariate analysis were performed using conditional logistic regression. The number of cases ≤ 5 in the independent variable group was analyzed by accurate logistic regression or properly merged. As well, we considered *p* < 0.05 to indicate a statistically significant test result for all of our statistical tests.

## 3. Results

### 3.1. General Information in the Case and Control Groups

Among the 140 pairs of subjects in the study, the mean age was 74.12 ± 16.35 years, ranging from 43 to 89 years. Most of these participants (about 63.57%) were married or widowed. In total, 257 (91.80%) subjects had only primary schooling or below. The average household income (per year) of 218 (77.90%) subjects was 20,000 RMB or less, and most subjects worked as fishermen (43.21%) or house-workers (40.36%). The average height was 159.79 ± 8.3 cm, and the average weight was 60.45 ± 9.38 kg. The average weight in the case group was 60.49 ± 10.93 kg, which was significantly higher than that in the control group, and this difference was statistically significant. Other detailed information is shown in Table 1.

### 3.2. Desalinated Water Intake and Calcium and Magnesium Concentrations in Urine

We found no significant difference in the desalinated water intake percentage between the case and control groups (*χ*2 = 0.032, *p* > 0.05, see Table 2). The mean concentrations of urinary calcium and magnesium were also not statistically different between the two groups (*t* = 0.399, *p* > 0.05 and *t* = 0.527, *p* > 0.05, see Table 3). Additionally, when grouped by desalinated water intake or no such intake, we observed no significant association between the calcium and magnesium levels in urine between the two groups (*t* = −0.59, *p* >0.05 and *t* = 0.31, *p* >0.05, see Table 4).

### 3.3. CVDs Risk Factors

Twenty-one risk factors including location, marital status, educational, and economic status were included in the univariate logistic regression analysis. This single-factor analysis showed that obesity, family history of hypertension, hypertension, diabetes, alcohol consumption, irritability, and depression were all risk factors for CVDs (*p* < 0.05) (see Table 5 and Table 6)

Table 7 shows the information on the OR of CVD risk factors using stepwise regression to filter variables and taking multivariate conditional logistic regression and precise logistic regression to analyze the data. From this, we found that obesity was an adverse factor for CVDs. Compared with the normal BMI group, the obesity group was associated with a 5.38 times greater chance of getting CVDs (OR = 5.38, 95% CI: 1.05–7.45, *p* = 0.04). History of hypertension had negative correlation with CVDs (OR = 3.61, 95% CI: 1.58–8.25, *p* < 0.01), and drinking status and irritability were also inversely associated with CVDs (OR = 2.57, 95% CI: 1.02–6.47, *p* = 0.05 and OR = 4.30, 95% CI: 1.93–9.60, *p* < 0.01). Nevertheless, physical activity had a significantly positive impact on CVDs (OR = 0.35, 95% CI: 0.16–0.75, *p* = 0.01).

## 4. Discussion

According to the Global Burden of Disease Study in 2017, the largest number of deaths from non-communicable diseases (NCDs) were estimated to come from CVDs, and ischemic heart disease and stroke accounted for 84.9% of all CVDs deaths. More specifically, ischemic heart disease and stroke ranked as the first and the third, respectively, in terms of years of life lost (YLL) in 2017 [20]. Recently, the prevalence of CVDs in China has begun to increase, and some studies predict that the prevalence may grow from 2018 to 2025 [21].

The lack of traditional freshwater resources has always been an important problem faced by China as well as many other parts of the world. Seawater desalination technology can increase the total amount of available fresh water and effectively solve the problem of freshwater resource shortage in China. Compared with freshwater, desalinated water is less susceptible to precipitation which is unevenly distributed in regions during the whole year. However, water hardness significantly declines after the desalination process. As classified by calcium carbonate concentration, water with hardness lower than 75 mg/L is normally classified as soft and classified as hard when this concentration is higher than 75 mg/L. Moreover, from the distillation or reverse osmosis processes, the level of calcium and magnesium in water can decline to varying degrees, and the content can become extremely low. The World Health Organization has indicated that hard water may reduce the risk of certain CVDs, possibly due to the protective effect of calcium and magnesium ions on the cardiovascular system. However, this conclusion is still controversial and there is no corresponding causal inference evidence to support it [22].

In this paper, we found that there was no significant difference in urinary calcium and magnesium content between different levels of desalinated seawater intake, but these results were not consistent with those from Israel. Israeli studies have shown that in cities that use desalinated water as the source of water, serum magnesium in the body decreased significantly after desalinated water intake, while in cities without desalinated water, serum magnesium in the body increased significantly during the same period [23]. Therefore, we infer that during the same period, the serum magnesium of the people who drank the desalinated water was significantly lower than that of the people who did not drink the desalinated water. We present the following reasons why the results of this study are different from those of the Israeli (and other) studies.

First, the different results may be due to the different detection methods and different biological samples selected by the different studies. Urinary magnesium and serum magnesium are both indices that measure the magnesium level in the body, but the half-life of magnesium concentration is different in different biological media. Serum magnesium generally measures the variation of magnesium over a long time such as several weeks or months, and urinary magnesium indicates the concentration range of magnesium during recent dietary intake [24]. Magnesium in the blood accounts for less than 1% of the whole body, so evaluating it by serum magnesium may not be the most accurate method [25]. However, some studies suggest that continuous monitoring of serum magnesium can effectively assess individual levels, although relatively stable excretion by urine shows the sufficiency of dietary magnesium [26]. Urinary magnesium is usually used to detect the absorption of magnesium in the intestinal tract, but a high variation of urinary magnesium may affect the test’s accuracy [27]. At present, there is no agreed-upon standard for detection method selection in CVDs studies, and there are few studies that focus on urinary magnesium and CVDs. Some studies have found that when urinary magnesium is at a low level, the risk and mortality of fatal and nonfatal ischemic heart disease are both increased, but no association between serum magnesium and the risk of ischemic heart disease has been found [28]. Compared with serum magnesium, the question of whether the association between urine magnesium and CVDs is more significant still needs further research, and this paper makes a small contribution to the knowledge of the relationship between urinary magnesium and CVDs.

Second, dietary habits are very important for mineral intake. In this study, we found no significant difference between urinary calcium and magnesium levels between our two groups. This may be because desalinated water intake had little effect on urinary calcium and magnesium levels in residents living in this area in general since drinking water is not the only way the body gets minerals; diet is more important. The subjects studied in this paper live in coastal areas, where seafood intake is quite large, and this may make up for the reduced intake of calcium and magnesium ions from desalinated seawater. In the future, adding a no seafood intake habits group might help to shed some light on this.

Third, accurate exposure assessments of desalinated water intake are very important. Here, in the process of investigation, we found that some residents still had doubts about the safety of desalinated seawater, and some residents thought that desalinated seawater has problems such as poor taste and no nutrition. In this study, only 37 subjects use the desalinated water as direct drinking water; 40 people used the desalinated seawater in indirect ways, such as cooking; and 203 people did not drink the desalinated water at all. One paper [29] in 2019 showed that only 9.7% of people in the county used desalinated water as the only source of drinking water and that 49.9% of people used desalinated water when cooking. Most people preferred to use desalinated water for washing or cleaning. In this study, the small population who chose desalinated water as the only water source may have led to selection bias.

At present, the drinking water standard for calcium and magnesium ion content in China is set forth in the *Standards for Drinking Water Quality* from 2006. Here, the total hardness is required to be less than 450 mg/L, and no lower limit is specified. There are some studies indicate that the total hardness generated by reverse osmosis technology is less than 75 mg/L, the concentration of magnesium is less than 7 mg/L, and the concentration of calcium is less than 6 mg/L [16,17], which are all lower than the *Hygienic Standard of Mineralization for Low-Mineral-Level Drinking Water*.

Our multivariate logistic regression analysis showed that obesity, hypertension, alcohol consumption, and irritability were all risk factors for CVDs, while physical activity was a positive factor against CVD risk. The previous work on CVDs has indicated that dyslipidemia of different obesity types over 35 years old in China is higher than that of the general population, and the *Report on Cardiovascular Health and Diseases in China 2020* showed that tobacco use, diet, physical activity, body weight, and psychology can affect CVD risk as well [30]. In addition, studies in Suzhou have shown that drinking alcohol more than 2 times a week can increase the risk of CVDs compared with non-drinking [31]. Analysis of high-risk factors for CVDs in Jilin Province [32] has also indicated that people who drink alcohol, and are overweight or obese are more likely to suffer from CVDs. The analysis also found that anxiety, irritability, and other adverse emotions can interact with the central nervous system and endocrine nervous system so as to bring about vasoconstriction, increase blood pressure and glucose levels, accelerate the progression of atherosclerosis, and even increase the risk of CHD [33]. Water accounts for about 70% of the total mass of the human body. According to the *Chinese Dietary Guidelines*, healthy adults need about 2500 mL of water every day. As a new source of fresh water to help alleviate the shortage of fresh water throughout parts of China and the rest of the world, the long-term effects of desalinated water on human health cannot be ignored. Our research suggests that further studies should therefore be carried out, especially those that can establish a cohort focused on the long-term effects of the calcium and magnesium levels in desalinated water on CVDs.

## 5. Conclusions

This nested case-control study does not provide evidence for an appreciable effect of desalinated water on the risk of CVDs. Any effect is likely to be extremely small and of considerably less risk than that of already-well-recognized cardiovascular risk factors. The results in this study clearly show that CVDs were related to unhealthy lifestyles, such as lack of physical activity, alcohol consumption, and negative mood. Future large prospective studies are required to examine the association between desalinated water and the risk of CVDs further. In addition, longer follow-up times are also needed to investigate the association between the level of calcium and magnesium in people living in different environments (such as heavy seafood eaters and no seafood eaters) and the incidence of CVDs.

## Figures and Tables

**Table 1:** Baseline characteristics in the case group and control group *.

Variable	Cases (n = 140)	Controls (n = 140)	Total	Statistic		*p*
				χ2	t	
*Marital status*				0.55		0.45
Married	92 (65.71%)	86 (61.43%)	178 (63.57%)			
Other	48 (34.29%)	54 (38.57%)	102 (36.43%)			
*Education*				0.43		0.51
Primary and below	130 (92.86%)	127 (90.71%)	257 (91.79%)			
Junior high school and above	10 (7.14%)	13 (9.29%)	23 (8.21%)			
*Occupation* ^Δ^				1.50		0.96
1	8 (5.71%)	9 (6.43%)	17 (6.07%)			
2	8 (5.71%)	6 (4.29%)	14 (5.00%)			
3	5 (3.57%)	6 (4.29%)	11 (3.93%)			
4	62 (44.29%)	59 (42.14%)	121 (43.21%)			
5	2 (1.43%)	2 (1.43%)	4 (1.43%)			
6	55 (39.29%)	58 (41.43%)	113 (40.36%)			
*Economic status (ten thousand RMB)* ^※^				2.91		0.41
<2	105 (75.00%)	113 (80.71%)	218 (77.86%)			
2-	25 (17.86%)	17 (12.14%)	42 (15.00%)			
5-	6 (4.29%)	5 (3.57%)	11 (3.93%)			
8-	4 (2.86%)	5 (3.57%)	9 (3.21%)			
*Height (cm)*	159.72 ± 7.53	159.96 ± 7.25	159.79 ± 8.30		0.33	0.75
*Weight (kg)*	60.49 ± 10.93	57.28 ± 9.66	60.45 ± 9.38		−3.07	<0.01 ^#^

* When the number of cases in the independent variable group was lower than 5, exact logistic regression was used. ^Δ^ Occupation code: 1. Administrative, enterprise, institution personnel, and related personnel; 2. Professional and technical personnel; 3. Business, catering, and service personnel; 4. Production personnel in agriculture, forestry, animal husbandry, fishing, and water conservancy industries; 5. Production and transportation equipment and related personnel; 6. Housework and leisure at home. ^※^ Economic status: average household income (per year). ^#^ Indicates a significant difference in the case group and control group.

**Table 2:** Desalinated water intake in the case group and control group.

Desalinated Water Intake	Cases (n = 140)	Controls (n = 140)	Total (n = 280)	*χ*2	*p*
Yes	19 (13.57%)	18 (12.86%)	37 (13.21%)	0.032	0.86
No	121 (86.43%)	122 (87.14%)	243 (86.79%)		

**Table 3:** Urinary calcium and magnesium levels in the case group and control group (mg/g creatinine).

Variable	Cases	Controls	Total	*t*	*p*
Calcium in urine	211.55 ± 145.86	200.41 ± 134.63	206.19 ± 137	0.399	0.53
Magnesium in urine	117.16 ± 62.59	119.80 ± 59.88	118.88 ± 31.3	0.527	0.47

**Table 4:** Urinary calcium and magnesium levels in different desalinated seawater intake groups (mg/g creatinine).

Variable	No Drink Desalinated Water		Drink Desalinated Water		*t*	*p*
	n	$\bar{x}\pm s$	n	$\bar{x}\pm s$		
Calcium in urine	203	205.00 ± 140.63	37	220.47 ± 126.15	−0.59	0.56
Magnesium in urine	203	119.94 ± 62.56	37	108.92 ± 40.74	1.31	0.20

**Table 5:** Univariate analysis of baseline information in CVDs *.

Variable	Cases (n = 140)	Controls (n = 140)	Wald *χ*2	OR	95% CI	*p*
*Location*						
H town	91 (65.00%)	104 (74.29%)				
G town	49 (35.00%)	36 (25.71%)	0.44	0.80	0.42–1.54	0.51
*Marital status*						
Married	92 (65.71%)	86 (61.43%)				
Other	48 (34.29%)	54 (38.57%)	2.26	1.69	0.83–3.35	0.13
*Education*						
Primary and below	130 (92.86%)	127 (90.71%)				
Junior high school and above	10 (7.14%)	13 (9.29%)	0.24	0.78	0.30–2.01	0.61
*Economic status (ten thousand RMB)*						
<2	105 (75.00%)	113 (80.71%)				
2-	25 (17.86%)	17 (12.14%)	0.81	1.39	0.68–2.87	0.37
5-	6 (4.29%)	5 (3.57%)	0.53	1.61	0.45–5.76	0.47
8-	4 (2.86%)	5 (3.57%)	1.08	0.42	0.08–2.17	0.30

* When the number of cases in the independent variable group was lower than 5, exact logistic regression was used.

**Table 6:** Univariate analysis of CVDs factors *.

Variable	Cases (n = 140)	Controls (n = 140)	Wald *χ*2	OR	95% CI	*p*
*Desalinated water intake*						
No	121 (86.43%)	122 (87.14%)				
Yes	19 (13.57%)	18 (12.86%)	0.03	0.94	0.49–1.83	0.87
*BMI*						
normal	85 (60.71%)	91 (65.00%)				
overweight	43 (30.71%)	41 (29.29%)	1.54	1.42	0.82–2.46	0.21
obesity	12 (8.57%)	8(5.71%)	4.58	3.60	1.11–11.64	0.03 ^#^
*Physical activity*						
No	91 (65.00%)	74 (52.86%)				
Yes	49 (35.00%)	66 (47.14%)	0.43	1.02	0.95–1.10	0.51
*Family history of Hypertension*						
No	114 (81.43%)	123 (87.86%)				
Yes	26 (18.57%)	17 (12.14%)	6.30	2.46	1.22–4.95	0.01^#^
*Family history of diabetes*						
No	136 (97.14%)	137 (97.86%)				
Yes	4 (2.86%)	3 (2.14%)	2.75	6.00	0.72–49.84	0.10
*History of Hypertension*						
No	46 (32.86%)	54 (38.57%)				
Yes	94 (67.14%)	86 (61.43%)	18.99	3.71	2.06–6.70	<0.01^#^
*History of diabetes*						
No	117 (83.57%)	119 (85.00%)				
Yes	23 (16.43%)	21 (15.00%)	6.00	2.75	1.22–6.18	0.01^#^
*History of hyperlipidemia*						
No	127 (90.71%)	132 (94.29%)				
Yes	13 (9.29%)	8 (5.71%)	3.70	4.50	0.97–20.83	0.05
*Health care products intake*						
No	111 (79.29%)	114 (81.43%)				
Yes	29 (20.71%)	26 (18.57%)	0.11	1.11	0.59–2.10	0.75
*Cooking oil consumption*						
≤30 g/day	76 (54.29%)	70 (50.00%)				
>30 g/day	64 (45.71%)	70 (50.00%)	1.32	0.74	0.45–1.23	0.25
*Salt consumption*						
≤10 g/day	100 (71.43%)	105 (75.00%)				
>10 g/day	40 (28.57%)	35 (25.00%)	0.02	0.96	0.55–1.68	0.89
*Smoking status*						
Never	93 (66.43%)	85 (60.71%)				
Ever	13 (9.29%)	16 (11.43%)	1.81	1.92	0.74–4.98	0.18
Current	34 (24.29%)	39 (27.86%)	0.90	1.49	0.65–3.39	0.34
*Second-hand smoke exposure*						
hardly	106 (75.71%)	112 (80.00%)				
Yes	34 (24.29%)	28 (20.00%)	0.13	0.88	0.43–1.73	0.71
*Alcohol drinking*						
No	92 (65.71%)	138 (98.57%)				
Yes	48 (34.29%)	29 (20.71%)	3.98	1.93	1.01–3.68	0.05 ^#^
*Tea drinking*						
No	128 (91.43%)	130 (92.86%)				
Yes	12 (8.57%)	10 (7.14%)	0.21	1.15	0.63–2.10	0.65
*Irritability*						
No	79 (56.43%)	100 (71.43%)				
Yes	61 (43.57%)	40 (28.57%)	19.13	4.60	2.32–9.11	<0.01 ^#^
*Anxious*						
No	127 (90.71%)	129 (92.14%)				
Yes	13 (9.29%)	11 (7.86%)	1.28	1.71	0.68–4.33	0.26
*Depression*						
No	104 (74.29%)	113 (80.71%)				
Yes	36 (25.71%)	27 (19.29%)	5.33	2.07	1.12–3.83	0.02 ^#^

* When the number of cases in the independent variable group was lower than 5, exact logistic regression was used. ^#^ Indicates a significant difference in the case group and control group and as risk factor.

**Table 7:** Multivariate logistic analysis of CVD factors.

Variable	Wald *χ*2	OR	95% CI	*p*
*BMI*				
Obesity vs. normal	4.09	5.38	1.05–27.45	0.04
*Physical activity*				
Yes vs. No	7.22	0.35	0.16–0.75	0.01
*History of hypertension*				
Yes vs. No	9.28	3.61	1.58–8.25	<0.01
*Alcohol drinking*				
Yes vs. No	3.98	2.57	1.02–6.47	0.05
*Irritability*				
Yes vs. No	12.08	4.30	1.93–9.60	<0.01

## Data Availability

The datasets used and analyzed in this study are available from the corresponding author upon reasonable request.
